# Poly[[triaqua­(μ_3_-4-oxidopyridine-2,6-dicarboxyl­ato)terbium(III)] monohydrate]

**DOI:** 10.1107/S1600536811005447

**Published:** 2011-02-23

**Authors:** Dong-Yu Lv, Zhu-Qing Gao, Jin-Zhong Gu

**Affiliations:** a Key Laboratory of Nonferrous Metal Chemistry and Resources Utilization of Gansu Province, College of Chemistry and Chemical Engineering, Lanzhou University, Lanzhou, Gansu 730000, People’s Republic of China; b School of Chemistry and Biology Engineering, Taiyuan University of Science and Technology, Taiyuan 030021, People’s Republic of China

## Abstract

In the title coordination polymer, {[Tb(C_7_H_2_NO_5_)(H_2_O)_3_]·H_2_O}_*n*_, the Tb^III^ atom is eight-coordinated by a tridentate 4-oxidopyridine-2,6-dicarboxyl­ate trianion, two adjacent monodentate anions and three water mol­ecules, forming a distorted bicapped trigonal–prismatic TbNO_7_ coordination environment. The anions bridge adjacent Tb^III^ ions into double chains. Adjacent chains are further connected into sheets parallel to (10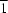). O—H⋯O hydrogen bonds involving both coordinated and uncoordinated water mol­ecules generate a three-dimensional network.

## Related literature

For structures and properties of luminescent lanthanide coordination compounds, see: Kustaryono *et al.* (2010 ▶); He *et al.* (2010 ▶); Li *et al.* (2008 ▶); Luo *et al.* (2008 ▶). For the use of multi-carboxyl­ate and heterocyclic acids in coordination chemistry, see: Li *et al.* (2008 ▶); Luo *et al.* (2008 ▶). For the dicarboxyl­ate ligand 4-oxido-pyridine-2,6-dicarboxyl­ate, see: Gao *et al.* (2008 ▶). For the isotypic structures of the Dy and Eu analogues, see: Gao *et al.* (2006 ▶) and Lv *et al.* (2010 ▶), respectively. For bond lengths and angles in other complexes with eight-coordinate Tb^III^, see: Chen *et al.* (2008 ▶); Ramya *et al.* (2010 ▶).
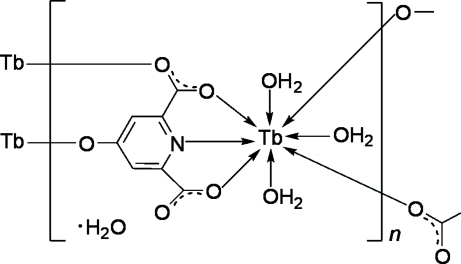


## Experimental

### 

#### Crystal data

[Tb(C_7_H_2_NO_5_)(H_2_O)_3_]·H_2_O
*M* *_r_* = 411.08
Monoclinic, 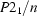

*a* = 9.953 (2) Å
*b* = 7.5454 (16) Å
*c* = 15.461 (3) Å
β = 105.126 (2)°
*V* = 1120.9 (4) Å^3^
*Z* = 4
Mo *K*α radiation
μ = 6.35 mm^−1^
*T* = 293 K
0.30 × 0.25 × 0.22 mm

#### Data collection

Bruker APEXII CCD diffractometer
Absorption correction: multi-scan (*SADABS*; Bruker, 2004 ▶) *T*_min_ = 0.162, *T*_max_ = 0.247
7828 measured reflections
2080 independent reflections
1929 reflections with *I* > 2σ(*I*)
*R*_int_ = 0.032

#### Refinement

*R*[*F* ^2^ > 2σ(*F* ^2^)] = 0.019
*wR*(*F* ^2^) = 0.051
*S* = 1.10
2080 reflections
196 parameters
12 restraints
H atoms treated by a mixture of independent and constrained refinement
Δρ_max_ = 1.34 e Å^−3^
Δρ_min_ = −0.60 e Å^−3^

### 

Data collection: *APEX2* (Bruker, 2004 ▶); cell refinement: *SAINT* (Bruker, 2004 ▶); data reduction: *SAINT*; program(s) used to solve structure: *SHELXS97* (Sheldrick, 2008 ▶); program(s) used to refine structure: *SHELXL97* (Sheldrick, 2008 ▶); molecular graphics: *SHELXTL* (Sheldrick, 2008 ▶) and *DIAMOND* (Brandenburg & Putz, 2005 ▶); software used to prepare material for publication: *publCIF* (Westrip, 2010 ▶).

## Supplementary Material

Crystal structure: contains datablocks I, global. DOI: 10.1107/S1600536811005447/wm2449sup1.cif

Structure factors: contains datablocks I. DOI: 10.1107/S1600536811005447/wm2449Isup2.hkl

Additional supplementary materials:  crystallographic information; 3D view; checkCIF report

## Figures and Tables

**Table 1:** Selected bond lengths (Å)

Tb1—O5^i^	2.3035 (19)
Tb1—O8	2.368 (2)
Tb1—O6	2.383 (2)
Tb1—O4^ii^	2.4106 (19)
Tb1—O3	2.415 (2)
Tb1—O7	2.416 (2)
Tb1—O1	2.424 (2)
Tb1—N1	2.471 (2)

Symmetry codes: (i) 
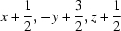
; (ii) 
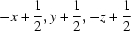
.

**Table 2:** Hydrogen-bond geometry (Å, °)

*D*—H⋯*A*	*D*—H	H⋯*A*	*D*⋯*A*	*D*—H⋯*A*
O6—H1*W*⋯O1^iii^	0.85 (4)	2.10 (3)	2.799 (3)	139 (3)
O6—H2*W*⋯O5^iv^	0.86 (4)	1.93 (3)	2.725 (3)	154 (3)
O7—H3*W*⋯O9^v^	0.88 (2)	1.84 (2)	2.687 (3)	162 (4)
O7—H4*W*⋯O9	0.85 (4)	2.23 (3)	2.995 (4)	151 (3)
O8—H5*W*⋯O2^vi^	0.85 (2)	1.85 (2)	2.693 (3)	175 (4)
O8—H6*W*⋯O3^ii^	0.85 (4)	1.85 (4)	2.680 (3)	167 (4)
O9—H7*W*⋯O2^vii^	0.86 (4)	1.84 (2)	2.699 (3)	175 (4)
O9—H8*W*⋯O4^i^	0.85 (4)	2.37 (4)	3.073 (4)	141 (5)

Symmetry codes: (i) 
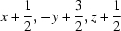
; (ii) 
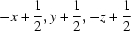
; (iii) 
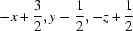
; (iv) 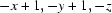; (v) 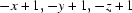; (vi) 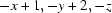; (vii) 
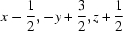
.

**Fig. 1.**
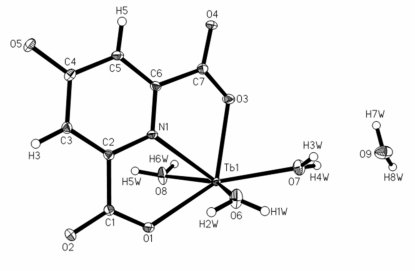
Drawing of the asymmetric unit of the title compound, showing the atom-labelling scheme. Displacement ellipsoids are drawn at the 30% probability level.

**Fig. 2.**
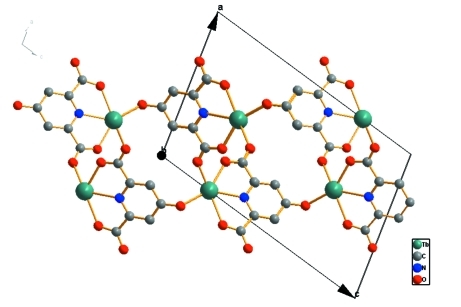
View along the b axis of the title compound, showing the double chain.

**Fig. 3.**
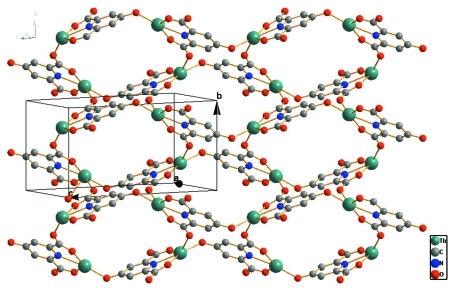
View approximately along the a axis, showing the sheet structure of {[Tb(C7H2NO5)(H2O)3].H2O}.

## supplementary crystallographic information

### Comment

The design and synthesis of luminescent lanthanide coordination polymers have achieved great progress during the last years owing to their potential applications in biomedical imaging, as luminescent sensors or as fluorescent probes (Kustaryono *et al.*, 2010; He *et al.*, 2010). Eu and Tb are the most useful lanthanides due to their good fluorescence properties (Li *et al.*, 2008; Luo *et al.*, 2008). Many multi-carboxylate or heterocylic carboxylic acids are used for designing such materials (Li *et al.*, 2008; Luo *et al.*, 2008). For lanthanide coordination polymers, 4-hydroxy-pyridine-2,6-dicarboxylic acid is an excellent bridging pyridine dicarboxylate ligand (Lv *et al.*, 2010; Gao *et al.*, 2008), which can provide at most one nitrogen atom and five O coordination sites. In order to extend the investigation in this field, we synthesized the lanthanide coordination polymer {[Tb(C_7_H_2_NO_5_)(H_2_O)_3_]^.^H_2_O}, and report its structure here.

The title compound is isotypic with its Dy (Gao *et al.*, 2006) and Eu (Lv *et al.*, 2010) analogues. As shown in Fig.1, the asymmetric unit contains one Tb(III) ion, one 4-oxidopyridine-2,6-dicarboxylate anion, three coordinated water molecules, and one water molecule of crystallisation. The Tb atom is eight-coordinated by seven oxygen atoms from three anions and three coordinated water molecules and by one nitrogen atom from one tridentate anion (the other two anions are monodentate), forming a distorted bicapped trigonal-prismatic coordination environment.

Important bond distances and angles are presented in Table 1. The Tb—O bond lengths [2.3035 (19) to 2.424 (2) Å] are shorter than the Tb—N bond length [2.471 (2) Å], which is in agreement with the bond lengths observed in other Tb(III) complexes (Chen *et al.*, 2008; Ramya *et al.*, 2010). The anion adopts a *µ*_3_-pentadentate coordination mode, as shown in Fig. 1. The anions bridge adjacent Tb^III^ ions to form infinite double chains (Fig. 2). Adjacent chains are further connected by the coordination of the anions and Tb(III) ions into a two-dimensional sheet parallel to (101) (Fig. 3), which are further extended into a three-dimensional framework through O—H···O hydrogen-bonding interactions including both coordinated and uncoordinated water molecules (Table 2).

### Experimental

To a solution of terbium(III) nitrate hexahydrate (0.136 g, 0.3 mmol) in water (5 ml) was added an aqueous solution (5 ml) of the ligand (0.060 g, 0.3 mmol) and a drop of triethylamine. The reactants were sealed in a 25-ml Teflon-lined stainless-steel Parr bomb. The bomb was heated at 433 K for 3 days. The cool solution contained single crystals in *ca* 60% yield. Anal. Calcd for C_7_H_10_TbNO_9_: C, 20.45; H, 2.45; N, 3.41. Found: C, 20.16; H, 2.17; N, 3.74.

### Refinement

The coordinated water H atoms were located in a different Fourier map and refined with distance constraints of O–H = 0.83 (3) Å. The free water H atoms were placed at calculated positions and refined with a riding model, considering the position of oxygen atoms and the quantity of H atoms. The carbon-bound H atoms were placed in geometrically idealized positions, with C—H = 0.93 Å and constrained to ride on their respective parent atoms, with *U*iso(H) = 1.2 *U*eq(C). The two highest remaining electron denstity peaks greater than one electron per Å^3^ are located at (0.4907 0.8249 0.2004) and (0.5006 0.8231 0.3054), repectively. The corresponding distances to the nearest atom (heavy atom Tb1) are *ca* 0.80 Å.

### Crystal data

**Table:**

[Tb(C_7_H_2_NO_5_)(H_2_O)_3_]·H_2_O	*F*(000) = 784
*M**_r_* = 411.08	*D*_x_ = 2.436 Mg m^−^^3^
Monoclinic, *P*2_1_/*n*	Mo *K*α radiation, λ = 0.71073 Å
Hall symbol: -P 2yn	Cell parameters from 5700 reflections
*a* = 9.953 (2) Å	θ = 2.2–28.3°
*b* = 7.5454 (16) Å	µ = 6.35 mm^−^^1^
*c* = 15.461 (3) Å	*T* = 293 K
β = 105.126 (2)°	Block, colorless
*V* = 1120.9 (4) Å^3^	0.30 × 0.25 × 0.22 mm
*Z* = 4	

### Data collection

**Table:**

Bruker APEXII CCD diffractometer	2080 independent reflections
Radiation source: fine-focus sealed tube	1929 reflections with *I* > 2σ(*I*)
graphite	*R*_int_ = 0.032
φ and ω scans	θ_max_ = 25.5°, θ_min_ = 2.2°
Absorption correction: multi-scan (*SADABS*; Bruker, 2004)	*h* = −12→11
*T*_min_ = 0.162, *T*_max_ = 0.247	*k* = −9→8
7828 measured reflections	*l* = −18→18

### Refinement

**Table:**

Refinement on *F*^2^	Secondary atom site location: difference Fourier map
Least-squares matrix: full	Hydrogen site location: inferred from neighbouring sites
*R*[*F*^2^ > 2σ(*F*^2^)] = 0.019	H atoms treated by a mixture of independent and constrained refinement
*wR*(*F*^2^) = 0.051	*w* = 1/[σ^2^(*F*_o_^2^) + (0.0227*P*)^2^ + 0.941*P*] where *P* = (*F*_o_^2^ + 2*F*_c_^2^)/3
*S* = 1.10	(Δ/σ)_max_ = 0.001
2080 reflections	Δρ_max_ = 1.34 e Å^−^^3^
196 parameters	Δρ_min_ = −0.60 e Å^−^^3^
12 restraints	Extinction correction: *SHELXL97* (Sheldrick, 2008), Fc^*^=kFc[1+0.001xFc^2^λ^3^/sin(2θ)]^-1/4^
Primary atom site location: structure-invariant direct methods	Extinction coefficient: 0.0244 (6)

### Special details

**Table:**

Geometry. All e.s.d.'s (except the e.s.d. in the dihedral angle between two l.s. planes) are estimated using the full covariance matrix. The cell e.s.d.'s are taken into account individually in the estimation of e.s.d.'s in distances, angles and torsion angles; correlations between e.s.d.'s in cell parameters are only used when they are defined by crystal symmetry. An approximate (isotropic) treatment of cell e.s.d.'s is used for estimating e.s.d.'s involving l.s. planes.
Refinement. Refinement of *F*^2^ against ALL reflections. The weighted *R*-factor *wR* and goodness of fit *S* are based on *F*^2^, conventional *R*-factors *R* are based on *F*, with *F* set to zero for negative *F*^2^. The threshold expression of *F*^2^ > σ(*F*^2^) is used only for calculating *R*-factors(gt) *etc*. and is not relevant to the choice of reflections for refinement. *R*-factors based on *F*^2^ are statistically about twice as large as those based on *F*, and *R*- factors based on ALL data will be even larger.

### Fractional atomic coordinates and isotropic or equivalent isotropic displacement parameters (Å^2^)

**Table:**

	*x*	*y*	*z*	*U*_iso_*/*U*_eq_	
Tb1	0.499147 (11)	0.823001 (18)	0.253250 (7)	0.01168 (10)	
C1	0.5913 (3)	0.8297 (4)	0.06235 (18)	0.0145 (6)	
C2	0.4542 (3)	0.7331 (4)	0.03360 (17)	0.0137 (6)	
C3	0.3928 (3)	0.6833 (4)	−0.05345 (18)	0.0157 (6)	
H3	0.4367	0.7069	−0.0984	0.019*	
C4	0.2628 (3)	0.5961 (4)	−0.07393 (17)	0.0150 (6)	
C5	0.2059 (3)	0.5615 (4)	−0.00148 (17)	0.0166 (6)	
H5	0.1218	0.5012	−0.0109	0.020*	
C6	0.2739 (3)	0.6163 (4)	0.08284 (17)	0.0149 (6)	
C7	0.2160 (3)	0.5940 (4)	0.16200 (17)	0.0176 (6)	
H1W	0.685 (3)	0.519 (5)	0.3081 (14)	0.033 (10)*	
H2W	0.678 (4)	0.555 (5)	0.2179 (17)	0.049 (12)*	
H3W	0.507 (4)	0.547 (2)	0.394 (2)	0.049 (13)*	
H4W	0.477 (4)	0.706 (4)	0.433 (2)	0.039 (12)*	
H5W	0.378 (3)	1.090 (5)	0.1149 (12)	0.032 (10)*	
H6W	0.339 (4)	1.121 (5)	0.196 (2)	0.051 (13)*	
H7W	0.348 (2)	0.664 (5)	0.544 (3)	0.051 (14)*	
H8W	0.457 (5)	0.756 (7)	0.608 (3)	0.11 (2)*	
N1	0.3970 (3)	0.7017 (3)	0.10190 (15)	0.0144 (5)	
O1	0.6315 (2)	0.8714 (3)	0.14414 (12)	0.0198 (5)	
O2	0.6576 (2)	0.8598 (3)	0.00587 (13)	0.0232 (5)	
O3	0.2780 (2)	0.6745 (3)	0.23225 (14)	0.0273 (6)	
O4	0.1088 (2)	0.5009 (3)	0.15414 (12)	0.0222 (5)	
O5	0.1971 (2)	0.5510 (3)	−0.15625 (12)	0.0191 (5)	
O6	0.6360 (2)	0.5619 (3)	0.25964 (14)	0.0286 (5)	
O7	0.5014 (3)	0.6624 (3)	0.38879 (14)	0.0254 (5)	
O8	0.3974 (3)	1.0761 (3)	0.17129 (14)	0.0294 (6)	
O9	0.4360 (3)	0.6777 (3)	0.5670 (2)	0.0376 (6)	

### Atomic displacement parameters (Å^2^)

**Table:**

	*U*^11^	*U*^22^	*U*^33^	*U*^12^	*U*^13^	*U*^23^
Tb1	0.00900 (13)	0.01520 (14)	0.01068 (12)	−0.00006 (5)	0.00229 (7)	−0.00069 (4)
C1	0.0132 (15)	0.0153 (15)	0.0153 (13)	0.0025 (11)	0.0043 (11)	0.0017 (10)
C2	0.0126 (15)	0.0137 (14)	0.0153 (12)	0.0009 (12)	0.0044 (11)	0.0017 (11)
C3	0.0146 (16)	0.0195 (16)	0.0137 (13)	0.0016 (11)	0.0047 (11)	0.0008 (10)
C4	0.0133 (15)	0.0159 (15)	0.0145 (12)	0.0045 (12)	0.0013 (10)	−0.0021 (10)
C5	0.0121 (15)	0.0184 (15)	0.0186 (13)	−0.0034 (12)	0.0030 (11)	−0.0012 (11)
C6	0.0113 (14)	0.0165 (15)	0.0168 (13)	−0.0007 (12)	0.0036 (11)	0.0023 (11)
C7	0.0136 (15)	0.0219 (16)	0.0170 (13)	0.0004 (13)	0.0035 (11)	0.0018 (11)
N1	0.0099 (13)	0.0182 (13)	0.0148 (11)	−0.0016 (10)	0.0027 (9)	−0.0013 (9)
O1	0.0145 (11)	0.0288 (12)	0.0164 (10)	−0.0054 (9)	0.0048 (8)	−0.0030 (8)
O2	0.0189 (12)	0.0352 (13)	0.0178 (10)	−0.0049 (10)	0.0087 (8)	0.0022 (9)
O3	0.0229 (13)	0.0446 (16)	0.0165 (10)	−0.0167 (10)	0.0087 (9)	−0.0084 (9)
O4	0.0184 (12)	0.0307 (13)	0.0178 (9)	−0.0134 (10)	0.0050 (8)	−0.0016 (8)
O5	0.0155 (11)	0.0272 (12)	0.0126 (9)	0.0033 (9)	−0.0001 (8)	−0.0052 (8)
O6	0.0329 (14)	0.0328 (14)	0.0226 (11)	0.0189 (11)	0.0118 (10)	0.0066 (10)
O7	0.0338 (15)	0.0226 (14)	0.0218 (11)	0.0025 (10)	0.0107 (10)	0.0009 (9)
O8	0.0394 (15)	0.0339 (14)	0.0198 (11)	0.0178 (11)	0.0163 (10)	0.0098 (10)
O9	0.0222 (15)	0.0273 (15)	0.0595 (18)	0.0038 (11)	0.0041 (13)	0.0031 (12)

### Geometric parameters (Å, °)

**Table:**

Tb1—O5^i^	2.3035 (19)	C5—C6	1.367 (4)
Tb1—O8	2.368 (2)	C5—H5	0.9300
Tb1—O6	2.383 (2)	C6—N1	1.347 (4)
Tb1—O4^ii^	2.4106 (19)	C6—C7	1.492 (4)
Tb1—O3	2.415 (2)	C7—O4	1.256 (3)
Tb1—O7	2.416 (2)	C7—O3	1.257 (3)
Tb1—O1	2.424 (2)	O4—Tb1^iii^	2.4106 (19)
Tb1—N1	2.471 (2)	O5—Tb1^iv^	2.3035 (19)
C1—O2	1.245 (3)	O6—H1W	0.85 (4)
C1—O1	1.263 (3)	O6—H2W	0.86 (4)
C1—C2	1.508 (4)	O7—H3W	0.875 (16)
C2—N1	1.345 (4)	O7—H4W	0.85 (4)
C2—C3	1.377 (4)	O8—H5W	0.849 (16)
C3—C4	1.412 (4)	O8—H6W	0.85 (4)
C3—H3	0.9300	O9—H7W	0.86 (4)
C4—O5	1.315 (3)	O9—H8W	0.85 (4)
C4—C5	1.405 (4)		
			
O5^i^—Tb1—O8	99.83 (8)	C3—C2—C1	123.8 (2)
O5^i^—Tb1—O6	85.81 (8)	C2—C3—C4	119.5 (3)
O8—Tb1—O6	148.11 (7)	C2—C3—H3	120.2
O5^i^—Tb1—O4^ii^	81.52 (7)	C4—C3—H3	120.2
O8—Tb1—O4^ii^	70.97 (7)	O5—C4—C5	121.4 (3)
O6—Tb1—O4^ii^	140.77 (7)	O5—C4—C3	122.2 (2)
O5^i^—Tb1—O3	151.44 (7)	C5—C4—C3	116.4 (2)
O8—Tb1—O3	93.13 (9)	C6—C5—C4	120.1 (3)
O6—Tb1—O3	96.50 (8)	C6—C5—H5	120.0
O4^ii^—Tb1—O3	78.83 (7)	C4—C5—H5	120.0
O5^i^—Tb1—O7	82.37 (8)	N1—C6—C5	123.3 (3)
O8—Tb1—O7	140.75 (8)	N1—C6—C7	113.5 (2)
O6—Tb1—O7	70.95 (8)	C5—C6—C7	123.2 (3)
O4^ii^—Tb1—O7	70.65 (7)	O4—C7—O3	124.5 (3)
O3—Tb1—O7	71.68 (8)	O4—C7—C6	118.9 (2)
O5^i^—Tb1—O1	80.00 (7)	O3—C7—C6	116.5 (3)
O8—Tb1—O1	74.95 (7)	C2—N1—C6	117.4 (2)
O6—Tb1—O1	75.22 (8)	C2—N1—Tb1	121.61 (19)
O4^ii^—Tb1—O1	137.48 (7)	C6—N1—Tb1	120.69 (18)
O3—Tb1—O1	128.19 (7)	C1—O1—Tb1	124.88 (18)
O7—Tb1—O1	142.73 (8)	C7—O3—Tb1	124.41 (18)
O5^i^—Tb1—N1	143.47 (8)	C7—O4—Tb1^iii^	138.84 (17)
O8—Tb1—N1	77.25 (8)	C4—O5—Tb1^iv^	127.69 (17)
O6—Tb1—N1	79.77 (8)	Tb1—O6—H1W	123 (2)
O4^ii^—Tb1—N1	129.18 (8)	Tb1—O6—H2W	114 (3)
O3—Tb1—N1	64.24 (7)	H1W—O6—H2W	112 (3)
O7—Tb1—N1	122.96 (8)	Tb1—O7—H3W	124 (2)
O1—Tb1—N1	63.95 (7)	Tb1—O7—H4W	125 (2)
O2—C1—O1	124.7 (3)	H3W—O7—H4W	110 (3)
O2—C1—C2	119.1 (2)	Tb1—O8—H5W	127 (2)
O1—C1—C2	116.2 (2)	Tb1—O8—H6W	109 (3)
N1—C2—C3	123.2 (3)	H5W—O8—H6W	115 (3)
N1—C2—C1	112.9 (2)	H7W—O9—H8W	114 (3)

Symmetry codes: (i) *x*+1/2, −*y*+3/2, *z*+1/2; (ii) −*x*+1/2, *y*+1/2, −*z*+1/2; (iii) −*x*+1/2, *y*−1/2, −*z*+1/2; (iv) *x*−1/2, −*y*+3/2, *z*−1/2.

### Hydrogen-bond geometry (Å, °)

**Table:**

*D*—H···*A*	*D*—H	H···*A*	*D*···*A*	*D*—H···*A*
O6—H1W···O1^v^	0.85 (4)	2.10 (3)	2.799 (3)	139 (3)
O6—H2W···O5^vi^	0.86 (4)	1.93 (3)	2.725 (3)	154 (3)
O7—H3W···O9^vii^	0.88 (2)	1.84 (2)	2.687 (3)	162 (4)
O7—H4W···O9	0.85 (4)	2.23 (3)	2.995 (4)	151 (3)
O8—H5W···O2^viii^	0.85 (2)	1.85 (2)	2.693 (3)	175 (4)
O8—H6W···O3^ii^	0.85 (4)	1.85 (4)	2.680 (3)	167 (4)
O9—H7W···O2^ix^	0.86 (4)	1.84 (2)	2.699 (3)	175 (4)
O9—H8W···O4^i^	0.85 (4)	2.37 (4)	3.073 (4)	141 (5)

Symmetry codes: (v) −*x*+3/2, *y*−1/2, −*z*+1/2; (vi) −*x*+1, −*y*+1, −*z*; (vii) −*x*+1, −*y*+1, −*z*+1; (viii) −*x*+1, −*y*+2, −*z*; (ii) −*x*+1/2, *y*+1/2, −*z*+1/2; (ix) *x*−1/2, −*y*+3/2, *z*+1/2; (i) *x*+1/2, −*y*+3/2, *z*+1/2.
